# Safety and Effectiveness of the New Generation APERIO® Hybrid Stent-retriever Device in Large Vessel Occlusion Stroke

**DOI:** 10.1007/s00062-021-01122-1

**Published:** 2021-12-09

**Authors:** Marius L. Vogt, Alexander M. Kollikowski, Franziska Weidner, Marc Strinitz, Jörn Feick, Fabian Essig, Hermann Neugebauer, Karl Georg Haeusler, Mirko Pham, Alexander Maerz

**Affiliations:** 1 Department of Diagnostic and Interventional Neuroradiology, University Hospital Würzburg, Würzburg, Germany; 2 Department of Neurology, University Hospital Würzburg, Würzburg, Germany

**Keywords:** Mechanical thrombectomy, Stent-retriever device, Stroke, APERIO, APERIO Hybrid

## Abstract

**Background:**

It is unknown whether technological advancement of stent-retriever devices influences typical observational indicators of safety or effectiveness.

**Methods:**

Observational retrospective study of APERIO**® **(AP) vs. new generation APERIO**®** Hybrid (APH) (Acandis®, Pforzheim, Germany) stent-retriever device (01/2019–09/2020) for mechanical thrombectomy (MT) in large vessel occlusion (LVO) stroke. Primary effectiveness endpoint was successful recanalization eTICI (expanded Thrombolysis In Cerebral Ischemia) ≥ 2b67, primary safety endpoint was occurrence of hemorrhagic complications after MT. Secondary outcome measures were time from groin puncture to first pass and successful reperfusion, and the total number of passes needed to achieve the final recanalization result.

**Results:**

A total of 298 patients with LVO stroke who were treated by MT matched the inclusion criteria: 148 patients (49.7%) treated with AP vs. 150 patients (50.3%) treated with new generation APH. Successful recanalization was not statistically different between both groups: 75.7% for AP vs. 79.3% for APH; *p* = 0.450. Postinterventional hemorrhagic complications and particularly subarachnoid hemorrhage as the entity possibly associated with stent-retriever device type was significantly less frequent in the group treated with the APH: 29.7% for AP and 16.0% for APH; *p* = 0.005; however, rates of symptomatic hemorrhage with clinical deterioration and in domo mortality were not statistically different. Neither the median number of stent-retriever passages needed to achieve final recanalization, time from groin puncture to first pass, time from groin puncture to final recanalization nor the number of cases in which successful recanalization could only be achieved by using a different stent-retriever as bail-out device differed between both groups.

**Conclusion:**

In the specific example of the APERIO**®** stent-retriever device, we observed that further technological developments of the new generation device were not associated with disadvantages with respect to typical observational indicators of safety or effectiveness.

## Introduction

Mechanical thrombectomy (MT) has become the standard of care for large vessel occlusion (LVO) in acute ischemic stroke [1–3]. In 2015 there were 5 large randomized trials (MR CLEAN, ESCAPE, REVASCAT, SWIFT PRIME and EXTEND IA) that first proved the efficacy of MT, each trial mainly employing stent-retriever devices to achieve recanalization [4–8]. Hence, current consensus statements and/or guidelines advocate the use of stent-retriever devices, which continues to be the predominant endovascular technical approach to achieve successful recanalization in LVO stroke [1, 2]. The most widely practiced techniques utilize stent-retriever embolectomy as the central procedural step of MT supported by one of several possible techniques of proximal occlusion/aspiration and/or distal aspiration. Subsequently, first randomized controlled trials tested distal aspiration alone, not with the aim to test whether it was superior to completely dispense with the use of a stent-retriever device but for raising evidence that this alternative probably is non-inferior [9, 10]. To date, a clear superiority of any single technique or device does not seem to emerge. There is a wide selection of different stent retriever devices available from different manufacturers. Over the last few years medical progress of endovascular stroke treatment has been paralleled by dynamic innovations in stent-retriever device technology. This led to both novel devices and also further technical refinements of first generation devices, such as modifications in material composition and their structural design. These advancements in medical device technology and engineering also represent potential improvements for interventional handling and also seem promising to have positive impact on recanalization effectiveness and safety; however, these assumptions need to be tested by studies with observational design and/or registries. Our retrospective analysis addressed this question by investigating whether stent-retriever device evolution would be associated with alterations in typical observational endpoints of safety or effectiveness in the specific example of two consecutive generations of one particular stent-retriever device (APERIO®, Acandis®, Pforzheim, Germany).

## Methods

### Patient Selection

We retrospectively included all consecutive patients with acute symptomatic LVO stroke who were treated by MT at our comprehensive stroke center (University Hospital Würzburg, Germany) between January 2019 and September 2020, using either the APERIO**®** (AP) or the APERIO**®** Hybrid (APH) stent-retriever device. Our institution serves as a center for teleneurological and teleradiological stroke medicine, including diagnosis, assessment of eligibility for MT, coordination of referral and subsequent MT with 24 h/7 day access to the angiography suite in a regional network in north-western Bavaria [11]. Informed consent was waived with approval by the local ethics committee. Occlusions of both the anterior (middle cerebral artery, MCA, anterior cerebral artery, ACA, distal internal carotid artery, ICA) and posterior (basilar artery, BA, vertebral artery, VA) circulations in the setting of acute ischemic stroke were taken into account.

### Clinical and Radiological Assessment

Clinical stroke severity as assessed by the National Institutes of Health Stroke Scale (NIHSS [12]) was evaluated by trained neurologists before (baseline) and after endovascular treatment (follow-up after 24, 48 and 72 h). All patients underwent a non-contrast computed tomography (CT) scan, CT angiography (CTA) and, if indicated, additional CT perfusion prior to endovascular treatment. Radiological evaluation of non-contrast CT scans was done by trained neuroradiologists using the Alberta Stroke Program Early Computed Tomography Score (ASPECTS) before and after MT for LVO in the anterior circulation [13]. Collateral status was evaluated for CT and digital subtraction angiography (DSA) imaging using both the score of Miteff et al. for CTA [14] and the score of the American Society of Interventional and Therapeutic Neuroradiology/Society of Interventional Radiology (ASITN/SIR) for DSA [15]. Endovascular treatment was evaluated using the expanded thrombolysis in cerebral infarction (eTICI) score [16], the total number of stent-retriever passes needed as well as the time intervals from groin to first pass and to final eTICI (in minutes). Primary safety endpoints were hemorrhagic complications after MT. To account for substantial heterogeneity in how symptomatic intracranial hemorrhage (sICH) is defined in the literature, we proceeded in the following manner: for all patients we applied the morphological classification of bleeding events according to the Heidelberg bleeding classification (HBC) [17], which is broadly consistent with the commonly used European cooperative acute stroke study (ECASS) II classification of hemorrhagic complications [18]. Ratings were performed by board-certified neuroradiologists and advanced neuroradiology fellows (AM, MS). We finally applied different definitions of sICH: first, we used the ECASS II definition of concomitant occurrence of any extravascular blood in the brain or within the cranium and clinical worsening (≥ 4 points on the NIHSS) [19], which seems to overestimate bleeding complications. Secondly, we defined sICH in accordance with the Safe Implementation of Thrombolysis in Stroke-Monitoring Study (SITS-MOST [20]) as concomitant occurrence of clinical worsening (≥ 4 points on the NIHSS) and evidence of hemorrhage rated PH2 (blood clots in more than 30% of the infarcted area with substantial space-occupying effect, according to ECASS), which is congruent with class 2, according to HBC. In order to differentiate reperfusion-related hemorrhage or hemorrhage into pre-existing parenchymal damage from potentially device attributable hemorrhage, we hypothesized that local subarachnoid hemorrhage (SAH) could possibly be associated with mechanical impact and thus be influenced by the specific device. It was our primary intention to investigate such possible influences by observation. As a novelty and with the aim to address this specific question, we defined possibly device-attributable symptomatic hemorrhage as the simultaneous occurrence of clinical worsening and CT-graphically detectable local subarachnoid hemorrhage (=symptomatic subarachnoid hemorrhage, sSAH) after MT. According to Liebeskind et al. successful reperfusion was defined as eTICI ≥ 2b67 in final angiographic control [21].

### Device Selection

At our institution the new generation APH thrombectomy device was introduced for MT in 09/2019, thus replacing its first-generation predecessor (AP). During the period of observation, the first-generation AP device was employed during 01/2019 to 04/2020. There was an overlapping interval of 6 months in which both stent-retriever devices were available and where the decision to use one of these two devices was at the discretion of the neurointerventionalist.

### APERIO® and the New Generation APERIO® Hybrid Stent-retriever Device

**Fig. 1:**
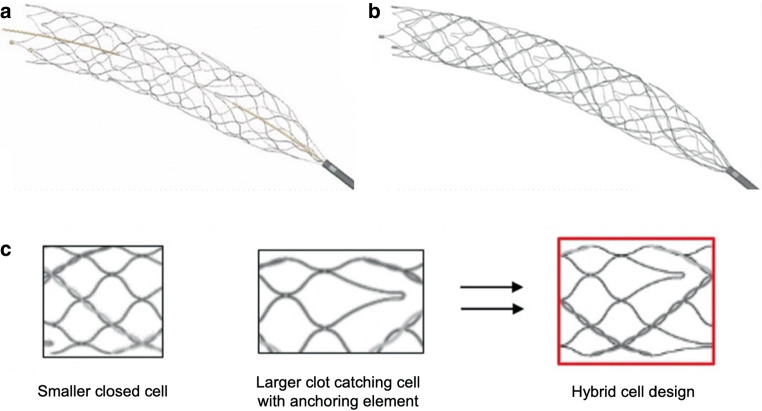
Illustration of the APERIO® stent-retriever device (**a**), the APERIO® Hybrid stent-retriever device with hybrid cell design (**b**) and zoomed-in picture of the hybrid design elements (**c**), provided upon request by courtesy of Acandis®

Both devices feature a hybrid cell design with smaller closed cells for better wall apposition and expansion into the clot and larger open cells that enable integration into the thrombus. Integrated anchoring elements provide additional support for atraumatic clot retention (Fig. 1).

**Fig. 2:**
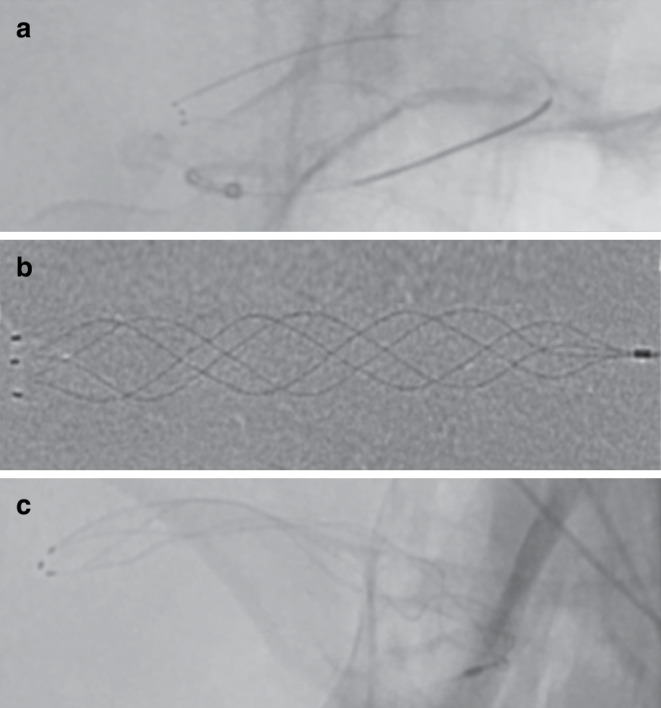
Fluoroscopic visibility of the deployed APERIO® stent retriever device (**a**) and the APERIO® Hybrid stent-retriever device ex vivo (**b**), provided upon request by courtesy of Acandis®, and the APERIO® Hybrid stent-retriever device deployed (**c**). Please note that unlike the AP, the APH is deployed without a distal wire tip and features substantially improved visibility over its full length with one proximal and three distal radiopaque markers

The new generation APH but not the first-generation AP features full-length visibility while maintaining its hybrid cell design. This was realized by braiding two radiopaque wires into the stent structure. These two wires are composed of nitinol and a platinum core to ensure elasticity and, at the same time, to optimize fluoroscopic visibility. Unlike the AP, the APH thrombectomy device works without a transport wire and without distal wire tip, which reaches through and beyond the stent-retriever in the first-generation AP device. The radiopaque marker concept of the APH is supplemented with one proximal and three distal radiopaque markers, which consist of platinum iridium for improved visualization in comparison to the gold markers of the AP (Fig. 2).

In order to evaluate the intraprocedural handling of both devices including aspects of visibility and navigability, a standardized questionnaire was answered by four participating neurointerventionalists. Visibility and tractability were retrospectively rated on a 4-point Likert scale (poor—neutral—good—very good).

The new generation APH comes in two additional sizes: 4.5 × 50 mm and 6.0 × 50 mm and has a longer transport wire of 212 cm compared to the AP (200 cm). Also, the radial force of the new generation APH is higher than the radial force of AP over all device sizes except the upper range of 4.5 × 30 mm.

### Procedure

All MT procedures were performed by board-certified neurointerventional experts or by supervised neurointerventional fellows in a biplane neuroangiography suite (Axiom Artis Q, Siemens Healthcare, Erlangen, Germany). All patients were under general anesthesia when treated. Endovascular access was obtained via transfemoral approach through the common femoral artery (CFA) using the Seldinger technique. In two cases transfemoral access to the target LVO lesion was not possible and required a transbrachial (*n* = 1) or transcervical (*n* = 1) access route. Nontherapeutic doses of 1000 IU unfractionated heparin were added to 1000 ml rinsing fluid (standard 0.9% saline solution) to avoid catheter-associated thrombus formation. A flexible aspiration catheter (e.g. SOFIA 5F or 6F, 125 cm length, MicroVention, Tustin, CA, USA) was biaxially advanced into the cervical segment of the internal carotid artery or the vertebral artery via guide catheter (e.g. Vista Brite tip, Cordis, Miami, FL, USA or IVA6F80, Balt, Montmorency, France). After visualization of the thromboembolic occlusion, a microcatheter (e.g. 21 or 27 Neuroslider®, Acandis) was advanced through the occluding thrombus over a microguidewire (e.g. Traxcess, MicroVention), a stent-retriever device was deployed and the aspiration catheter was placed in close proximal vicinity to the thrombus/embolus.

As per institutional standard, we applied stent-retriever deployment and stent-embolus retrieval supported by aspiration via an intermediate catheter advanced to the proximal end of the stent-retriever (supported also by manual aspiration at the proximal guiding catheter) [22, 23].

### Statistical Analysis

We used standard descriptive statistical measures including mean ± standard deviation (SD) or median with interquartile range (IQR) for numerical data and absolute or relative frequency distribution for categorical variables. Depending on the parametric or nonparametric distribution of data, intergroup comparison was performed using Student’s t‑test or Mann-Whitney U‑test for continuous variables, χ^2^-test or Fisher’s exact test for categorical variables. A two-sided *p*-value < 0.05 was considered statistically significant. Statistical analyses were performed using IBM SPSS Statistics for Windows (Version 26.0, IBM, Armonk, NY, USA).

## Results

### Baseline Characteristics

A total of 298 patients matched the inclusion criteria. The median age was 78 years (IQR 66–83 years), 54.0% were female and median baseline NIHSS was 14 (IQR 9–17). Of the patients 148 (56.1% female; median age: 78 years) underwent MT using the AP thrombectomy device vs. 150 patients (52.0% female; median age: 78 years) using the APH thrombectomy device. There were no significant differences in the following baseline variables: sex, age, NIHSS, cardiovascular risk factors (arterial hypertension, diabetes mellitus, hyperlipidemia, atrial fibrillation, positive family history, smoking) and baseline anticoagulation or antiplatelet therapy (Table 1). A total of 64 (43.2%) patients of the AP group vs. 55 (36.7%) patients of the APH group received intravenous alteplase prior to endovascular treatment (*p* = 0.246). The distribution of vessel occlusion sites differed slightly but statistically significantly as LVO in the anterior circulation was more frequent in the AP group (95.3% vs. 86.7% in the APH group; *p* = 0.009). Occlusion of the basilar artery was more frequent in the APH group (AP: 3.4% vs. APH: 10.7%; *p* = 0.014).

**Table 1:** Baseline demographics and characteristics of the study population and both groups treated with the APERIO® and the APERIO® Hybrid stent-retriever device

	APERIO®	APERIO® Hybrid	Total	*p*-value
*Age* in years—median (IQR)	78 (67–84)	78 (63–83)	78 (66–83)	0.588
*Sex—n (%)*				
Female	83/148 (56.1)	78/150 (52.0)	161/298 (54.0)	0.480
*Risk factors—n (%)*				
Arterial hypertension	118/145 (81.4)	113/139 (81.3)	231/284 (81.3)	0.985
Diabetes mellitus	36/145 (24.8)	33/139 (23.7)	69/284 (24.3)	0.831
Hyperlipidemia	57/145 (39.3)	54/137 (39.4)	111/282 (39.4)	0.986
Atrial fibrillation	63/145 (43.4)	54/137 (39.4)	117/282 (41.5)	0.492
*Pre-intervention anticoagulation—n (%)*	26/148 (17.6)	32/150 (21.3)	58/298 (19.5)	0.411
Phenprocoumon	8/148 (5.4)	11/150 (7.3)	19/298 (6.4)	0.496
NOAC	13/148 (8.8)	16/150 (10.7)	29/298 (9.7)	0.583
Heparin	5/148 (3.4)	5/150 (3.3)	10/298 (3.4)	0.900
*Pre-interventional antiplatelet therapy—n (%)*	42/148 (28.4)	30/150 (20.0)	72/298 (24.2)	0.091
ASS	36/148 (24.3)	24/150 (16.0)	60/298 (20.1)	0.073
Clopidogrel	2/148 (1.4)	4/150 (2.7)	6/298 (2.0)	0.684
Dual antiplatelet therapy	4/148 (2.7)	2/150 (1.3)	6/298 (2.0)	0.446
*Site of occlusion—n (%)*				
Anterior circulation	141/148 (95.3)	130/150 (86.7)	271/298 (90.9)	0.009
M1	56/148 (37.8)	66/150 (44.0)	122/298 (40.9)	0.279
M2	28/148 (18.9)	21/150 (14.0)	49/298 (16.4)	0.252
Distal ICA	57/148 (38.5)	42/150 (28.0)	99/298 (33.2)	0.054
ACA	0/148 (0.0)	1/150 (0.7)	1/298 (0.3)	n.a.
Basilar artery	5/148 (3.4)	16/150 (10.7)	21/298 (7.0)	0.014
PCA	2/148 (1.4)	4/150 (2.7)	6/298 (2.0)	0.684
*NIHSS baseline—median (IQR)*	14 (9–17)	14.5 (10–19)	14 (9.5–18)	0.236
*ASPECTS*				
Baseline—*median (IQR)*	8 (7–9)	8 (7–9)	8 (7–9)	0.010
ASPECTS 0–5—*n (%)*	2/141 (1.4)	16/130 (12.3)	18/271 (6.6)	–
ASPECTS 6–7—*n (%)*	43/141 (30.5)	41/130 (31.5)	84/271 (31.0)	–
ASPECTS 8–10—*n (%)*	96/141 (68.1)	73/130 (56.2)	169/271 (62.4)	–
*CTA collaterals *(MITEFF score)	2 (1–3)	2 (1–2)	2 (1–2)	0.394
*DSA collaterals* (ASITN/SIR)	2 (0–3)	1 (0–2)	2 (0–2)	0.282
*Intravenous alteplase—n (%)*	64/148 (43.2)	55/150 (36.7)	119/296 (40.2)	0.246

*NOAC* new oral anticoagulants, *ASS* acetylsalicylic acid, *M1/M2* middle cerebral artery, *ICA* internal carotid artery, *ACA* anterior cerebral artery, *PCA* posterior cerebral artery, *ASPECTS* Alberta Stroke Program Early Computed Tomography Score, *CTA* computed tomography angiography, *DSA* digital subtraction angiography, *ASITN* American Society of Interventional and Therapeutic Neuroradiology, *SIR* Society of Interventional Radiology

### Radiological/Angiographic Characteristics

Patients treated with the new generation APH had a statistically significantly lower ASPECT score in the CT scan prior to MT (*p* = 0.010) with nominal median ASPECT scores of 8 (IQR 7–9) for both groups. Collateral scores in both CT and conventional angiography were not significantly different in both groups. Stenting of ICA tandem stenosis at cervical level was necessary in 19 (12.8%) patients of the AP group compared to 6 (4.0%) patients of the APH group (*p* = 0.006). Intracranial bail-out stenting to preserve patency of underlying intracranial stenosis was performed in one single patient treated with the new generation APH thrombectomy device. Tractability and visibility of the AP and APH were retrospectively rated by 4 of our interventionalists. With regards to tractability, both the AP and the new APH were equally rated as “good” by one and “very good” by 3 of our 4 interventionalists. Visibility of the AP, on the other hand, was rated as “neutral” or “good” by 2 interventionalists each, while visibility of the new APH was rated as “very good” by all 4 participating neurointerventionalists.

### Effectiveness

Successful recanalization (eTICI ≥ 2b67) was achieved in 112 (75.7%) patients of the AP group and in 119 (79.3%) patients of the APH group, revealing no significant differences in recanalization rates between the two devices (*p* = 0.450). A median of 2 passages (IQR 1–3) were needed to achieve successful recanalization in both groups. In 8/150 (5.3%) patients treated with the new generation APH recanalization was not successful despite using different bail-out thrombectomy devices (vs. 2/148, 1.4%, patients in the AP group, *p* = 0.056). In 4/148 (2.7%) patients treated with the AP and in 6/150 (4.0%) patients treated with the new generation APH device secondary distal occlusions occurred within the same territory after thrombectomy of the primary target LVO lesion and were treated with secondary distal thrombectomy. In these cases, distal recanalization was possible using a smaller thrombectomy device (Catch Mini 3 × 15 mm, Balt, Montmorency, France) in 2/4 patients of the AP group and in 6/6 patients of the APH group. In 5/150 (3.3%) patients treated with the new generation APH device successful recanalization was achieved only after switching to another thrombectomy device vs. 3 (2.0%) treated with the AP. Mean time from in domo imaging to groin puncture, time from groin puncture to first pass with first flow restoration and time from groin to final successful recanalization was not statistically different between both groups (Table 2).

**Table 2:** Primary and secondary endpoints

	APERIO®	APERIO® Hybrid	Total	*p*-value
*Stent—PTA of cervical ICA tandem lesion—n (%)*	19/148 (12.8)	6/150 (4.0)	25/298 (8.4)	0.006
*Final eTICI—n (%)*				
0	2/148 (1.4)	9/150 (6.0)	12/298 (4.0)	0.033
1	6/148 (4.1)	4/150 (2.7)	10/298 (3.4)	0.506
2a	12/148 (8.1)	12/150 (8.0)	24/298 (8.1)	0.973
2b50	16/148 (10.8)	6/150 (4.0)	22/298 (55.7)	0.025
2b67	43/148 (29.1)	35/150 (23.3)	78/298 (7.4)	0.261
2c	30/148 (20.3)	36/150 (24.0)	66/298 (22.1)	0.438
3	39/148 (26.4)	48/150 (32.0)	87/298 (29.2)	0.284
*Successful recanalization (eTICI* *≥* *2b67)—n (%)*	112/148 (75.7)	119/150 (79.3)	231/298 (77.5)	0.450
*Time metrics, in min; mean ± SD; median (IQR)*				
In domo imaging to groin puncture	40.92 ± 18.15	40.58 ± 21.67	40.75 ± 19.97	0.890
	39 (28–49)	37 (26–50)	38 (28–49)	
Groin puncture to first pass	39.17 ± 21.54	42.35 ± 15.07	40.71 ± 18.69	0.161
	33 (24–51)	41 (31–50)	38 (28–50)	
Groin puncture to successful recanalization (eTICI **≥** 2b67)	86.45 ± 47.89	96.78 ± 47.18	91.55 ± 47.73	0.074
	78 (49–116)	91 (62–120)	88 (57–119)	
*Use of >* *1 device—n (%)*	11/148 (7.4)	16/150 (10.7)	27/298 (9.1)	0.331
Successful recanalization with different stent-retriever device	3/148 (2.0)	5/150 (3.3)	8/298 (2.7)	0.485
Successful recanalization with APERIO**®** as bail-out device	2/148 (1.4)	0/150 (0.0)	2/298 (0.7)	–
Peripheral fragmentation and use of smaller bail-out device	4/148 (2.7)	6/150 (4.0)	10/298 (3.4)	0.534
Unsuccessful despite bail-out-device	2/148 (1.4)	8/150 (5.3)	10/298 (3.4)	0.056
*Number of passages needed—median (IQR)*	2 (1–3)	2 (1–3)	2 (1–3)	0.306
*NIHSS—median (IQR)*				
Baseline	14 (9–17)	14.5 (10–19)	14 (9.5–18)	0.236
24 h	11 (6–22)	14 (5–22)	12 (5–22)	0.717
48 h	10 (4–18)	12 (5–21)	10 (4–19)	0.211
72 h	8 (3–17)	10 (3–20)	9.5 (3–19)	0.276
*ASPECTS*				
Baseline*—median (IQR)*	8 (7–9)	8 (7–9)	8 (7–9)	0.010
ASPECTS 0–5*—n (%)*	2/141 (1.4)	16/130 (12.3)	18/271 (6.6)	–
ASPECTS 6–7*—n (%)*	43/141 (30.5)	41/130 (31.5)	84/271 (31.0)	–
ASPECTS 8–10*—n (%)*	96/141 (68.1)	73/130 (56.2)	169/271 (62.4)	–
Follow-up*—median (IQR)*	7 (6–8)	7 (5–8)	7 (5–8)	0.387

*PTA* percutaneous transluminal angioplasty, *ICA* internal carotid artery, *eTICI expanded* thrombolysis in cerebral infarction, *NIHSS* National Institutes of Health Stroke Scale, *ASPECTS* Alberta Stroke Program Early Computed Tomography Score

### Safety

There was a significant difference in the rate of postinterventional hemorrhage and the stent-retriever used to perform MT, as postinterventional hemorrhage defined according to morphological classification of ECASS and HBC was less frequent in the APH group. Moreover, subarachnoid hemorrhage (HBC 3c) was significantly less frequent in the APH group (44/148, 29.7% vs. 24/150, 16.0%, *p* = 0.005). There was no statistical difference between both groups regarding clinical deterioration of ≥ 4 points on the NIHSS concomitant with either evidence of any form of postinterventional hemorrhage (as defined in ECASS II), evidence of PH2 (ECASS) or HBC type 2 hemorrhage (as defined in SITS-MOST) or evidence of subarachnoid hemorrhage (sSAH). In domo mortality of patients treated with the new generation APH was slightly higher (21/148, 14.2% vs. 28/150, 18.7%) but without significant difference (Table 3).

**Table 3:** Overview and classification of postinterventional hemorrhage and safety

		APERIO®	APERIO® Hybrid	Total	*p*-value
*ECASS*	*HBC*	*n* (%)	*n* (%)	*n* (%)	
0	0	87/148 (58.8)	111/150 (74.0)	198/298 (66.4)	0.005
HI1	1a	13/148 (8.8)	9/150 (6.0)	22/298 (7.4)	0.358
HI2	1b	19/148 (12.8)	13/150 (8.7)	32/298 (10.7)	0.245
PH1	1c	18/148 (12.2)	14/150 (9.3)	32/298 (10.7)	0.430
PH2	2	11/148 (7.4)	3/150 (2.0)	14/298 (4.7)	0.027
	3a (remote parenchyma)	1/148 (0.7)	1/150 (0.7)	2/298 (0.7)	0.992
	3b (intraventricular)	10/148 (6.8)	4/150 (2.7)	14/298 (4.7)	0.095
	3c (subarachnoid)	44/148 (29.7)	24/150 (16.0)	68/298 (22.8)	0.005
	3d (subdural)	0/148 (0.0)	0/150 (0.0)	0/298 (0.0)	n.a.
*sICH* = clinical deterioration ≥ 4 points (NIHSS)					
+ any hemorrhagic complication (in accordance with ECASS II)		25/148 (16.9)	22/150 (14.7)	47/298 (15.8)	0.598
+ PH 2 (ECASS)/2 (HBC) (in accordance with SITS-MOST)		5/148 (3.4)	1/150 (0.7)	6/298 (2.0)	0.096
+ subarachnoid hemorrhage (=sSAH 3c in accordance with HBC)		17/148 (11.5)	12/150 (8.0)	29/298 (9.7)	0.310
*In domo mortality*		21/148 (14.2)	28/150 (18.7)	49/298 (16.4)	0.297

*ECASS* European Cooperative Acute Stroke Study, *HBC* Heidelberg Bleeding Classification, *HI* Hemorrhagic Infarction, *PH* parenchymal hematoma, *sICH* symptomatic intracranial hemorrhage, *NIHSS* National Institutes of Health Stroke Scale

## Discussion

In this study both the first generation APERIO® (AP) and its successor, the new generation APERIO® Hybrid (APH) stent-retriever device achieved similarly high rates of successful recanalization (eTICI ≥ 2b67: 75.7% for AP vs. 79.3% for APH) according to the definition by Liebeskind et al. 2019 [21]. Full recanalization, defined as eTICI ≥ 2c, could be achieved in 46.7% for AP vs. 56.0% for APH. These observations did not significantly differ between the two devices but were nominally lower in the AP group. In both groups a median of 2 (IQR 1–3) passes were required for recanalization. Additionally, there were few more cases in the APH group (8 (5.3%) vs. 2 (1.4)) where successful recanalization could not be achieved despite using various bail-out devices. Hence, in these cases we believe the lack of successful recanalization cannot be related to the stent-retriever that has been employed.

Considering the different definitions of successful recanalization, our primary measures of effectiveness fall into comparable ranges of external estimates reported by large prospective registry studies like the North American STRATIS registry (87.9% full recanalization/mTICI ≥ 2b, median (IQR) of 1 (1–2) and 2 (1–3) passes, depending on stent-retriever size), the TRACK registry (80.3% full recanalization/mTICI ≥ 2b, mean and SD of 1.9 ± 1.2 passes) or the German Stroke Registry (83.0% full recanalization/mTICI ≥ 2b; median and IQR of 2 (1–3) passes) [24–28]. In view of our observations and their comparison with external estimates, both devices can be considered effective to achieve successful recanalization. This interpretation is further supported by the observation that in this study only 5 LVO (3.3%) in the APH group and only 3 LVO (2.0%) in the AP group eventually could not be recanalized using these stent-retriever as first choice but could be recanalized after switching to other types of stent-retriever.

In terms of safety, we looked at hemorrhagic complications after MT and focused on the hemorrhagic entity we considered to be possibly associated with the mechanical impact of the stent-retriever on the vessel wall: subarachnoid hemorrhage in spatial association with the site of mechanical endovascular manipulation. Regardless of the clinical neurological relevance of intracranial hemorrhage, there was a significant difference of the rates of postinterventional hemorrhage within 24 h between both groups as any intracranial hemorrhage occurred less frequently in the APH group. Interestingly, any signs of potentially device-attributable subarachnoid hemorrhage were in fact detected less frequently for the new generation APH device (24/150, 16.0%) compared with its predecessor (44/148, 39.7%). Obviously, it is of importance to include the clinical/neurological course of the patient when evaluating postinterventional hemorrhagic sequelae. To this end, there are various previously published definitions of symptomatic intracranial hemorrhage (sICH). In order to ensure proper comparability, we applied these pre-existing definitions of sICH also to our groups. According to the ECASS II protocol sICH is defined as any hemorrhage detected after MT that is associated with clinical deterioration of ≥ 4 points on NIHSS [19]. In SITS-MOST only parenchymal hematoma defined as PH2 according to ECASS or type 2 according to HBC, associated with clinical deterioration of ≥ 4 points on the NIHSS was defined as sICH [20]. By applying these two definitions of sICH we observed no significant differences between both groups. The prevalence of postinterventional sICH according to ECASS II was relatively high in our study (16.9% for AP vs. 14.7% for APH), but it needs to be acknowledged that the rates of hemorrhagic complications may be fairly overestimated using this definition, as a causal relationship between hemorrhagic transformation without mass-effect and clinical deterioration can only vaguely be inferred. By applying the sICH definition according to SITS-MOST, we observed considerably lower rates of sICH for both groups (3.4% for AP vs 0.7% for APH, *p* = 0.096). Although there was no statistically significant difference between both groups, sICH was remarkably low in the group treated with the new generation APH. Our sICH rates are in a range that is comparable to other external estimates. The TRACK registry reported rates of sICH (defined as any parenchymal, subarachnoid or intraventricular hemorrhage associated with worsening of the NIHSS by ≥ 4 points within 24 h) between 4.5–9.8%, depending on volume of stroke centers [29]. In the STRATIS registry the rate of sICH (defined as any PH1, PH2, remote intraparenchymal hemorrhage or intraventricular hemorrhage associated with worsening of the NIHSS by ≥ 4 points within 24 h) was reported to be 1.4% [26] and in a meta-analysis of the HERMES collaboration Goyal et al. reported 4.4% of postinterventional sICH (as defined by each trial) [3]. As we mentioned before, in our attempt to associate the occurrence of CT signs of hemorrhage to stent-retriever type, we had to define a novel type of sICH: CT detection of subarachnoid hemorrhage on postinterventional CT with concomitant clinical worsening of ≥ 4 points on NIHSS within 24 h (=sSAH). By applying this definition, we found no significant difference between both groups (11.5% for AP vs. 8.5% for APH). These results suggest that the new generation APH is efficient and safe in comparison to its predecessor with a trend towards improved safety with respect to hemorrhagic complications; however, the finding of the aforementioned difference in the detection of hemorrhage between groups may also represent the potential bias that considerably more patients of the AP group required stenting for tandem stenosis of the internal carotid artery. This usually resulted in double platelet inhibition immediately or soon after the intervention and hence leads to an increased risk of hemorrhage especially into predamaged ischemic infarction tissue, which we believe is independent from the stent retriever device used. Subjective ratings from interrogating four participating neurointerventionalists indicated that tractability was equally “good” or “very good” for both devices while visibility of the APH was rated as “very good” by all respondents and was improved compared to its predecessor. The substantially improved visibility of the new generation APH may also be at least partly held accountable for this favorable evolution of hemorrhagic risk to a very low level over time in this study, as it allows better control over the retrieval maneuver in the most critical moments of MT and substantially better visibility. Any potential bias underlying this interpretation cannot be completely resolved by this study. The observed estimates of bleeding type frequency that we found per group are descriptive in that these results cannot prove or disprove direct or indirect cause association with the stent-retriever type; however, with the intent of comparing two groups (novel device generation vs. predecessor generation) we found no evidence of substantial differences between bleeding type frequency, which supports the clinically relevant conclusion that the novel device type is equally safe with respect to bleeding complications that were comprehensively classified and evaluated with particular detail for the purpose of this study.

In domo mortality was at 14.2% for the AP group and at 18.7% for the APH group with the limitation that we did not collect any follow-up data after discharge. Therefore, the comparability with the observations of other studies and current registries is limited in this respect (mortality: STRATIS 14.4%, TRACK 19.8% and German Stroke Registry 28.5%) [24, 26, 27].

The observation of marginally fewer hemorrhagic complications in the group treated with the new generation APH did not result in a better early clinical outcome as assessed by the NIHSS at follow-up (24/38/72 h) and in-hospital mortality; however, the higher proportion of patients with basilar artery occlusion in the APH group must also be considered as a potential bias, as it has been described as an independent predictor of unfavorable outcome [30].

Procedural time metrics were not different between both stent-retriever groups, indicating that handling and navigability of the new generation APH is not inferior to the AP device. This comprises the median time interval from groin to first pass, which was slightly but not significantly shorter for the AP (33 min vs. 41 min) and which also was within a range that is comparable to other studies outside of the randomized controlled intervention trials. Pfaff et al., for example, reported a median of 35 min from groin to first flow restoration observing a new generation stent-retriever from another manufacturer [31]. There was also no statistical difference in the time from groin to final recanalization or in the time from groin to first pass.

In an already published early experience multicenter study of 48 patients, the APH stent-retriever device has been described as feasible, efficient and safe by Kaschner et al. [32]. Our study adds to this previous evidence by evaluating a substantially larger cohort and, in particular, providing an equally large and relatively well-balanced reference group treated with the previous model of the APERIO® stent-retriever device. We consider as strengths of our study that a relatively large number of patients could be observed and that both groups were similar in size and regarding baseline characteristics. For these reasons, we believe that despite its retrospective design, this study does not preclude the clinically relevant interpretations. At the same time, we acknowledge that the risk of any bias including also unidentified factors is inherently higher through retrospective observation. This includes also a possible increase of experience over time and its potential impact on the reported complication rates; however, by accurately reporting procedural time intervals we can provide one of the most important surrogate measures of operator experience. These measures suggest that there do not seem to be any relevant differences in operator experience. Arguably the most relevant strength of this study was that the transition between first and successive next generation devices was chronological at our institution with only a short period of negligible temporal overlap where both devices were available.

## Conclusion

The new generation APERIO**®** Hybrid thrombectomy device was observed to be efficient and safe in direct comparison to its predecessor. In addition, postinterventional hemorrhage, and particularly local subarachnoid hemorrhages as a bleeding entity possibly associated with the stent-retriever device occurred less frequently, which could be, at least in part, related to refinements of stent-retriever technology and improved handling and visibility of the new generation device.
